# Medical Image Classification Utilizing Ensemble Learning and Levy Flight-Based Honey Badger Algorithm on 6G-Enabled Internet of Things

**DOI:** 10.1155/2022/5830766

**Published:** 2022-05-29

**Authors:** Mohamed Abd Elaziz, Alhassan Mabrouk, Abdelghani Dahou, Samia Allaoua Chelloug

**Affiliations:** ^1^Faculty of Computer Science Engineering, Galala University, Suze 435611, Egypt; ^2^Artificial Intelligence Research Center (AIRC), Ajman University, Ajman 346, UAE; ^3^Department of Mathematics, Faculty of Science, Zagazig University, Zagazig 44519, Egypt; ^4^Mathematics and Computer Science Department, Faculty of Science, Beni-Suef University, Beni Suef 62511, Egypt; ^5^Mathematics and Computer Science Department, University of Ahmed DRAIA, Adrar 01000, Algeria; ^6^Department of Information Technology, College of Computer and Information Sciences, Princess Nourah bint Abdulrahman University, P.O. Box 84428, Riyadh 11671, Saudi Arabia

## Abstract

Recently, the 6G-enabled Internet of Medical Things (IoMT) has played a key role in the development of functional health systems due to the massive data generated daily from the hospitals. Therefore, the automatic detection and prediction of future risks such as pneumonia and retinal diseases are still under research and study. However, traditional approaches did not yield good results for accurate diagnosis. In this paper, a robust 6G-enabled IoMT framework is proposed for medical image classification with an ensemble learning (EL)-based model. EL is achieved using MobileNet and DenseNet architecture as a feature extraction backbone. In addition, the developed framework uses a modified honey badger algorithm (HBA) based on Levy flight (LFHBA) as a feature selection method that aims to remove the irrelevant features from those extracted features using the EL model. For evaluation of the performance of the proposed framework, the chest X-ray (CXR) dataset and the optical coherence tomography (OCT) dataset were employed. The accuracy of our technique was 87.10% on the CXR dataset and 94.32% on OCT dataset—both very good results. Compared to other current methods, the proposed method is more accurate and efficient than other well-known and popular algorithms.

## 1. Introduction

Providing medical diagnoses in real time using modern communication technologies such as the sixth-generation wireless communications standard (6G) is a major topic. Fortunately, early diagnosis of infections that affect sensitive human body sections may assist in restricting disease transmission and safeguarding the afflicted body parts (e.g., cancers spread rapidly throughout the body). Without a proper and timely diagnosis, illnesses may spread rapidly, resulting in a high risk of death [1, 2]. For instance, pneumonia diagnosis and prediction are difficult in medical imaging and healthcare, which are still under research. The fast growth of medical devices, communication technology, cloud computing, and the Internet of Medical Things (IoMT) may improve healthcare. The IoMT is a series of Internet-connected devices that aid in medical operations and activities [3]. The IoMT and 6G technology recently gave the medical field new tools/approaches to enhance illness detection and provide a rapid medical diagnosis. Furthermore, a vast quantity of data, such as computed tomographic (CT) scans, is created daily, showing the challenge of analyzing these images in real time to aid in the early detection of disease. Medical images may be restricted to researchers in the medical field alone due to privacy issues. As a result, the medical field continues to face hurdles in categorizing medical images. Furthermore, CT scans are low-resolution, noisy, and complex to analyze, posing difficulties in terms of disease detection accuracy.

Compared to earlier approaches for early diagnosis, the 6G-enabled IoMT offers a framework for analyzing multiple slices of CT scans in real time, allowing for speedy and reliable replies. Furthermore, various deep learning (DL) models and architectures were developed for the IoMT infrastructure integration [4, 5]. For instance, the MobileNet architecture is a widely used model which can be easily integrated into embedded systems with low resource. In addition, the MobileNet [6] models showed remarkable performance in terms of medical image analysis and disease diagnosis assistance [7, 8]. The recently developed DL models such as DenseNet [9], ShuffleNet [10], NASNet [11], and EfficientNet [12] rely on transfer learning which boost their performance based on using pretrained models on large amount of domain-specific data rather than using the same untrained model on new data [13, 14]. The employment of DL became one of the most popular and widely applied algorithms for computer vision tasks, such as improving loss function [15], feature selection optimization [16], and information interaction perception network [17]. Furthermore, most of the DL models applied to image analysis are based on convolutional neural networks (CNNs) which are widely used in medical imaging as robust feature extractors. However, the usage of these models can be costly in terms of the model size, number of extracted and learned features (representation space), and the computational complexity (time). In addition, the models are not easily integrated into IoT devices, which is a major challenge for the medical field. Thus, developing an optimized framework for the 6G IoT-enabled medical images is promising for the medical field especially when applying optimization algorithms to only select the most relevant extracted features from a DL model and reduce the representation space and boost the system performance. In addition, single DL models are less challenging in terms of development than ensemble-based DL models, whereas the latest one can learn more meaningfully features from image data which can be used to improve the system performance [18].

Recently, the feature selection (FS) methods have been developed to improve the performance of the classification task [16, 19]. For diagnosing medical images, in [20], they proposed a modified crow search algorithm as an FS technique to improve Parkinson's disease. This algorithm has been established its performance against other models. In [21], an opposition-based crow search (OCS) algorithm has been introduced as FS to determine the relevant features extracted using the DL model. This model has been used to classify brain image, lung cancer, and Alzheimer's disease in IoMT environment. With the advantages of these FS methods that achieved to improve the performance of classification in IoMT, they still have some limitations such as stuck in local optima and slow convergence towards the optimal solution. In addition, according to the none-free lunch theory, the optimization algorithm cannot solve all the optimization problems (such as FS) with the same efficiency. This motivated us to developed an alternative FS model for IoMT environment using a modified new meta-heuristic technique named honey badger algorithm (HBA) [22].

In general, the HBA emulates the behaviour of honey badger in nature to catch its food. According to these behaviours, HBA has been applied to solve different optimization and engineering problems as illustrated in [22]. Also, it has been applied to identify the parameter of proton-exchange membrane fuel cells as in [23] and to improve the sidelobe suppression in the antenna radiation patterns as in [24].

Therefore, this paper proposes a system for improving diagnostic imaging recognition effectiveness in terms of classification accuracy, which will be incorporated into the 6G-enabled IoMT. The system is composed of parts including feature extraction and feature selection. In the first part, a DL-based ensemble architecture was implemented incorporating two well-known DL models named MobileNet and DenseNet. At this stage, the ensemble model is trained to learn and extract more complex and meaningful image representation which will be fed to the FS part. In the second part, a novel FS algorithm is proposed by improving HBA relying on the Levy flight (LF) algorithm to reduce the representation space of medical images and boost the model performance. Using two real-world datasets, a complete evaluation of the proposed methodology is provided and compared to several state-of-the-art works.

The main contributions of this work are summarized as follows:

1. We propose an ensemble DL-based model to extract features from medical images collected using IoT devices.
2. We proposed an FS algorithm named Levy flight-based honey badger algorithm (LFHBA) where the operators of the Levy flight were used to improve the honey badger algorithm's capabilities.
3. The proposed FS algorithm aims to select the most relevant features from the extracted image representations which maximize the classification precision and accuracy.
4. The proposed framework can be integrated in a 6G-enabled IoMT system to reduce human intervention in medical facilities and provide quick diagnostic results.

According to the paper's structure, Section 2 reviews recent works on medical imaging and its applications. In Section 3, we provide the background of honey badger algorithm (HBA) and Levy flight (LF).  Section 4 offers a detailed description of our proposed 6G-enabled IoMT framework.  Section 5 lists the outcomes of image recognition experiments conducted to validate the proposed framework. Lastly, the conclusion and future direction are presented in Section 6.

## 2. Related Works

The transfer learning (TL) approach has become increasingly popular in recent years, which improves the efficiency of the models, reduces financial costs, and does not require more input data [25, 26]. Recently, TL was used for extracting features to solve the problems of the traditional deep learning methods (for more information, see [27]). Extracting features from VGG and ResNet networks, combining bilinear and classification algorithms, and learning them with SVM classifiers produced the best outcomes [28]. Esteva et al. [29] employed a mixture of data-driven technologies and the InceptionV3 to train dermatological images, achieving great results on the test set that were equivalent to professional dermatologists. Yu et al. [30] proposed a deep residual network-based phased classification technique, and segmentation was used to classify disease. However, it is not a complete solution because the ultimate classification must be done step by step [31]. Guo et al. [32] developed a multi-CNN using the adaptive sample learning technique to replicate intra-class disagreement and related noise interference.

Instead of developing a CNN from scratch with randomly initialized parameters, researchers used a pretrained CNN and transfer learning to classify medical images throughout the whole dataset [33]. As a result of this pretraining, CNN's training time was greatly reduced, resulting in an accuracy rate of 84.8% over five categories. In this case, transfer learning helps models trained on one task or large dataset after gaining a large knowledge to be transferred and applied to different but related tasks. Lopez et al. [34] used a deep learning-based technique to detect disease early. To address the medical image classification task, they used a modified VGGNet architecture and a transfer learning methodology. On the ISIC Archive dataset, the proposed technique had a sensitivity value of 78.66%. In a study by Ayan and Ünver [35], among an extended and nonaugmented dataset, the effectiveness of CNN model for identification of medical images was evaluated. However, they stated that when there is not enough data, deep learning methods could be useful [15]. Using the enhanced dataset, the network had a greater classification accuracy than just the model that did not.

CNNs have been widely used in medical image analysis in recent years due to their robust features representation abilities and have shown significant improvements. Yu et al. [30] suggested a multi-stage system for automatic disease recognition in medical images based on an extremely deep residual network. When SVM classifiers were used to collect high-level features from VGG and ResNet networks for bilinear merging, Ge et al. [28] achieved some of the greatest recognition results on a range of test sets. Following [36], they designed an aggregation of multi-level fully convolutional networks. A multi-CNN collaborative class label lesion recognition framework was designed by Zhang et al. [37]. Their method was more robust of lesion identification, and its usefulness had been tested using relevant data. A robust ensemble architecture, constructed using dynamic classifier selection techniques, was employed to detect cancer [38], so that the model can learn more powerful and distinguishing features. A crossnet-based combination of various convolutional networks was suggested as a solution for medical image identification [39] and proven by extensive testing. MobileNet and DenseNet were combined to create a lightweight and efficient classification model [40]. Different from prior methods, they used the well-known classification approach in the lightweight classification algorithm to increase feature discrimination, to decrease computational cost, and to keep the number of parameter values to a minimum.

Recently, Internet of Medical Things (IoMT) technology has proven to be ideal for constructing smart systems capable of accurately diagnosing illness in the same way that specialists do. According to [7], IoMT technology has aided in the creation of critical medical systems. Doctors can now get it in a range of locations, with greater patient diagnostic capacity without influencing subjective aspects. The issue of unbalanced data between unusual and widespread diseases, on the other hand, remained an unresolved challenge for any framework. As a result, this issue resulted in poor performance. In the medical profession, however, the classifier must be certain of its accuracy with a large proportion when detecting the type of cancer. According to a prior research, accurate detection is crucial for providing patients with the appropriate treatment. As a result, we are attempting to improve medical diagnostics.

A wide variety of real-world complicated optimization issues have been successfully solved using meta-heuristic algorithms. Due to their ability to utilize a list of candidate solutions rather than a single solution, they are efficiently able to traverse the solution space. As a result, meta-heuristic algorithms outperform other optimization methods. Many meta-heuristic strategies have been developed to help schedule tasks in the IoMT [41]. Some of the existing FS methods suffer from premature convergence and local minima, especially when faced with a large solution space [42]. Often, this limit results in inefficient task scheduling solutions, which has a negative impact on system performance. A global optimal solution to the IoMT task scheduling problem is therefore urgently needed. Hence, this paper aims to find the best solutions that leads to an increase in the rate of convergence, as shown in the next sections.

## 3. Preliminaries

This section briefly describes the honey badger algorithm (HBA) and Levy flight (LF) algorithms used for selecting the most relevant features.

### 3.1. Honey Badger Algorithm

The honey badger algorithm (HBA) is a meta-heuristic optimization approach created by Hashim et al. [22]. HBA may be regarded as a universal optimization method since it includes both exploration and exploitation stages. The stages of the HBA are outlined mathematically as follows. The HBA starts by forming the initial population of *N* solutions (i.e., honey badgers) using the following equation [22].

(1) $$\begin{array}{c} x_{i}=lb_{i}+r_{1}\times \left(ub_{i}-lb_{i}\right), \end{array}$$

where *x*_*i*_ denotes the *i*th honey badger location. In the search domain, *lb*_*i*_ refers to the lower bound and *ub*_*i*_ refers to the upper bound. Furthermore, a randomised value between 0 and 1 is *r*_1_.

The next step is to compute the Intensity that is dependent on the prey's attention force and the range between the prey and the badger (*i*th). *I*_*i*_ represents the prey's scent intensity; if the scent is strong, the prey will move quickly, and vice versa, according to the inverse square law, as stated by the following equation [22].

(2) $$\begin{array}{c} I_{i}=r_{2}\times \frac{S}{4\pi d_{i}^{2}},\;S={\left(x_{i}-x_{i+1}\right)}^{2},d_{i}=x_{\text{prey}}-x_{i}, \end{array}$$

where *S* is the attention force, as *S*=(*x*_*i*_ − *x*_*i*+1_)^2^. *d*_*i*_ indicates the range between both the prey and the *i*th badger, as *d*_*i*_=*x*_prey_ − *x*_*i*_.

Thereafter, HBA is split into two steps: digging and honey. During the digging step, a badger adopts a cardioid form. The cardioid movement is formulated using the following equation [22]:

(3) $$\begin{array}{c} x_{\text{new}}=x_{\text{prey}}+F\times \beta \times I\times x_{\text{prey}}+F\times r_{3}\times \alpha \times d_{i}\times \left|\cos\left(2\pi r_{4}\right)\times \left[1-\cos\left(2\pi r_{5}\right)\right]\right|, \end{array}$$

where the density parameter (*α*) governs time-varying randomness by the following equation [22]:

(4) $$\begin{array}{c} \alpha =C\times \;\exp\left(\frac{-t}{t_{\text{max}}}\right), \end{array}$$

where *C* is a constant that is greater than or equal to 1. Also, *t*_max_ denotes the highest number of cycles. In equation (3), *x*_prey_ represents the location of the prey that has been discovered to be the optimum so far—the global optimum location. The honey badger's food-finding ability is *β* ≥ 1. (Default = 6) is the honey badger's food-finding ability. *r*_3_, *r*_4_, and *r*_5_ are three distinct randomly initialized parameters ranging from 0 to 1. *F* is a flag that changes the direction of the search; it is calculated by the following equation [22]:

(5) $$\begin{array}{c} F=\left\{\begin{array}{cc} 1, & \text{if }r_{6}\leq 0.5,\\ -1, & \text{otherwise}. \end{array}\right) \end{array}$$

Finally, equation (6) can be used when a badger follows a honey bee to arrive at a beehive [22].

(6) $$\begin{array}{c} x_{\text{new}}=x_{\text{prey}}+F\times r_{7}\times \alpha \times d_{i}, \end{array}$$

where *x*_new_ represents the badger's latest place, *x*_prey_ represents the prey position, and *F* and *α* are calculated using equation (4) and (5), respectively. Based on spatial knowledge of *d*_*i*_, it can be shown in equation (6) that a badger undertakes a search near to the prey position *x*_prey_ discovered so far. At this step, the search is influenced by time-varying search behaviour (*α*). Furthermore, a honey badger may discover *F* disturbance. The algorithm's pseudocode is described in Algorithm 1.

### 3.2. Levy Flight

Levy flight is a kind of chaotic system in which the magnitude of the leap is determined by the likelihood function. We employ the Levy flight as in [43] in our job. When a high fly identifies a prey region, Aquila determines the land and then strikes. This is known as contour flight with quick glide invasion. In this case, Aquila optimization closely investigates the target prey's specified region in preparing for the assault. This behaviour is expressed formally as follows [43].

(7) $$\begin{array}{c} x_{\text{new}}=x_{\text{prey}}\times \text{Levy}\left(D\right)+X_{R}\left(t\right)+\left(y-x\right)*\text{rand,} \end{array}$$

where *x*_new_ is the new position that is produced by the search technique (*x*). The dimensionality space is denoted by *D*, and the Levy flight distribution is denoted by Levy(*D*), which is derived using equation (8). At the *i*th cycle, *X*_*R*_(*t*) is a random value chosen from the range [1 *N*] [43].

(8) $$\begin{array}{c} \text{Levy}\left(D\right)=s\times \frac{u\times \sigma }{{\left|r\right|}^{1/p}}, \end{array}$$

where *s* is a constant set to 0.01, *u* is a randomised value between 0 and 1, and *r* is a randomised number between 0 and 1. Equation (9) is used to compute *σ* [43].

(9) $$\begin{array}{c} \sigma =\left(\frac{\Gamma \left(1+\beta \right)\times \text{sin}e\left(\pi \beta /2\right)}{\Gamma \left(1+\beta /2\right)\times \beta \times 2^{\left(\beta -1/2\right)}}\right), \end{array}$$

where *β* is a constant set to 1.5. *y* and *x* are being used to display the circular form in the seek in equation (7), and they are given by the following equation [43].

(10) $$\begin{array}{c} y=r\times \text{cos}\left(\theta \right),\\ x=r\times \text{sin}\left(\theta \right),\\ r=r_{1}+U\times D_{1},\\ \theta =-w\times D_{1}+\theta_{1},\\ \theta_{1}=\frac{3\times \pi }{2}. \end{array}$$

For a given number of search iterations, *r*_1_ denotes the value from 1 to 20, and *U* is a tiny value set to 0.00565. *D*_1_ represents integers ranging from 1 to Dim (i.e., the length of the search space), and *w* represents a tiny value set to 0.005.

## 4. Proposed Approach

**Figure 1:**
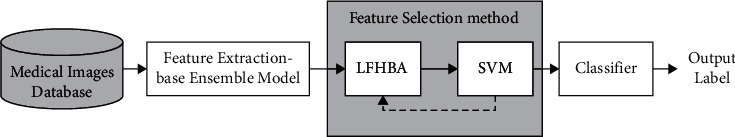
The proposed methodology.

To achieve our objective, we developed a 6G-enabled IoMT framework for medical image classification. Based on the principles of the 6G network and the DL architecture, we developed a technique that consists of three stages, as illustrated in Figure 1. (1) The first stage extracts the representation (features) of the input image using an ensemble DL model; (2) the second stage reduces the dimensionality of the extracted features by selecting the important features using a novel proposed feature selection algorithm based on improved honey badger algorithm and Levy flight (LFHBA); and (3) the selected features are fed into an ML classifier for classification tasks.

### 4.1. Feature Extraction Using Ensemble Deep Learning

**Figure 2:**
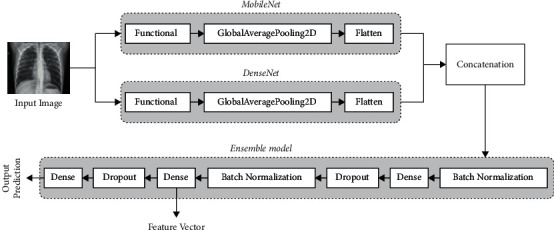
The structure of ensemble model block based on the extracted features of MobileNet and DenseNet.

This section describes the implemented DL architecture based on ensemble learning and transfers learning techniques. The objective of the developed model is to learn and extract medical image representation using two well-known DL models, including MobileNet and DenseNet. As shown in Figure 2, the input image to ensemble model is fed to two functional layers simultaneously. At this stage, each functional layer represents a pretrained model that relies on MobileNetV2 and DenseNet169, respectively. Each functional layer's output (learned representations) is fed to the global average pooling layer for dimensionality reduction. After applying the pooling operation on each parallel flow, the output is flattened and concatenated to generate a single feature vector of each inputted image. To fine-tune the overall network, overcome overfitting, and boost the classification accuracy, a sequential set of layers was placed on top of each other, including batch normalization (BN), fully connected layer (dense), and dropout layer as shown in Figure 2. The final output of the ensemble model is generated using a fully connected layer with a single output node to output the classification probability. Meanwhile, the dense layer before the final output is used to extract the learned image representations and feed them to the FS phase.

Using different image datasets, the ensemble model was fine-tuned to learn and extract feature vectors from inputted images of size 224 × 224. The DL models such as MobileNetV2 and DenseNet169 were pretrained on the ImageNet dataset [44]. In our experiments, the ensemble pretrained model was employed and fine-tuned on the datasets having chest and optical images. As an output, the MobileNetV2 and DenseNet169 generate a feature vector of size 1280 and 1664 after flattening, respectively. Thus, the concatenated feature vector is of size 2944. During the fine-tuning of the ensemble model, MobileNetV2 and DenseNet169 weights were fixed to accelerate the training process.

Meanwhile, the MobileNetV2 model building block consists of an inverted residual block core component, which is inspired by the bottleneck blocks. The inverted residual block contains two important blocks: the depthwise separable convolution block and skip connections used to link the input and output features on the same channels, thus improving the features representations with low memory usage. The depthwise separable convolution block consists of 3 × 3 depthwise convolution, BN, activation function, and 1 × 1 pointwise convolution where the order of execution of the layers is as follows: (3 × 3Conv)⟶(*BN*)⟶(ReLU)⟶(1 × 1Conv)⟶(*BN*)⟶(ReLU). Each building block can integrate a depthwise separable convolutional layer with different nonlinearity functions such as ReLU/ReLU6. Meanwhile, the DenseNet169 model has fewer parameters to be optimized and reduces the vanishing gradient problem in large models. DenseNet169 consists of 169 low parameters layers where each layer (*L*) is connected to every other layer with short connections (*L*(*L*+1)/2 connections).

To extract the feature vector from each input image, we used the generated fine-tuned model (model with the best classification accuracy) on each dataset. The extracted feature vector for each image of size 128 will be fed into the FS process in the proposed framework. The model was fine-tuned for 100 epochs with a batch of size 32 on each dataset to produce the best classification performance. Meanwhile, to update the model's weight and bias parameters, we used the RMSprop optimizer with a learning rate of 1*e* − 4. To overcome the model's overfitting, we used the dropout layer with a probability of 0.38 and data augmentation with the following transformations: random horizontal flip, random zoom, random width shift, random height shift, and random brightness.

### 4.2. The Enhanced HBA as FS Algorithm

**Figure 3:**
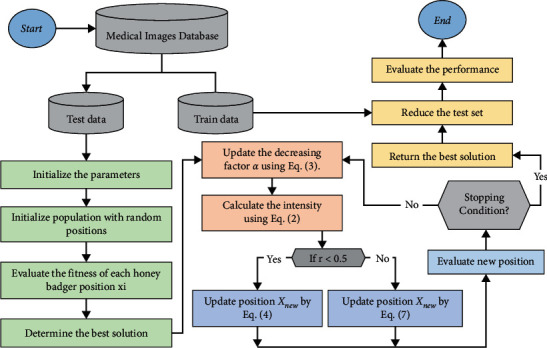
Flowchart showing the proposed algorithm.

The extracted features from the ensemble model are high-dimensional feature sets with 128 features that demand high computational complexity and may decrease the effectiveness of a classifier. As a result, these features are input into the feature selection (FS) step, which filters out duplicate and unnecessary features. As a result, a novel approach for improving the honey badger algorithm's (HBA) efficiency is presented in this study using Levy flight, which is employed to get more sustainable results. When the HBA is unable to obtain the best solution at the current epoch, a more effective search relying on Levy flight is performed to avoid being trapped in a locally optimal solution. The Levy flight search enhances the capacity to do both global and local searches at the same time.  Figure 3 illustrates the various phases of the developed FS approach.

To eliminate unnecessary and duplicated features, the developed FS approach employs a novel Levy flight-based honey badger algorithm (LFHBA). Initially, in the developed LFHBA, the beginning locations of each honey badger in the population are randomly assigned between [0, 1]. Each individual has a dimension equivalent to the total of features collected from the ensemble model. If there are *n* retrieved features and *N* badgers, for fitness calculation, each *x*_*i*_ would have a randomised value of 0 or 1 that is separated into either 1 or 0. The support vector machine (SVM) is used to compute the fitness value, which is the most commonly used for classification tasks [45], for a variety of reasons: the SVM is used to solve binary problems by maximizing the difference between both classes around a hyperplane. Hence, the ideal hyperplane is obtained with the greatest distance to the nearest training site of any class, resulting in an acceptable class distinction. Following the computation of the fitness value, the best solution is determined and the process of updating the solutions is conducted using the operators of LFHBA.

The developed LFHBA begins with the creation of a collection of *N* agents *X* that represent the FS problem solutions. To apply this process, the following equation is used:

(11) $$\begin{array}{c} X_{i}=\text{rand}\times \left(U-L\right)+L,\;i=1,2,\ldots ,N,\;j=1,2,\ldots ,\text{Dim}, \end{array}$$

where Dim refers to the number of features. As a consequence, the accessible dimensionality is limited to values ranging from *U* to *L*. Once we have determined the binary value of each *X*_*i*_ using equation (12), we can use it to figure out what it signifies.

(12) $$\begin{array}{c} BX_{ij}=\left\{\begin{array}{cc} 1, & \text{if }X_{ij}>0.5,\\ 0, & \text{otherwise}. \end{array}\right) \end{array}$$

We will next compute the fitness value of each *X*_*i*_ depending on its binary decision *BX*_*i*_, and the objective function is defined as

(13) $$\begin{array}{c} {\text{Fit}}_{i}=\lambda \times \gamma_{i}+\left(1-\lambda \right)\times \left(\frac{\left|BX_{i}\right|}{\text{Dim}}\right). \end{array}$$

In this situation, the fraction of relevant features is given as (|*BX*_*i*_|/Dim). SVM validation loss is *γ*_*i*_. Since SVM is more trustworthy and has lower complexity than other classification methods, SVM is often utilized. The parameter *λ* adjusts the proportion between both the efficiency of a classifier's forecasts and the feature selection.

The LF or HBA operators are employed in the proposed technique to modify solution *X*_*i*_. This is achieved by using the probability *P*_*i*_ connected with each *X*_*i*_. The LF will be employed if the probability of *P*_*i*_ is greater than 0.5, as stated by the following equations:

(14) $$\begin{array}{c} X_{ij}=\left\{\begin{array}{cc} \text{update }X_{i}\text{ using equation}\left(3\right), & \text{if }P_{i}<0.5,\\ \text{update }X_{i}\text{ using equation}\left(7\right), & \text{otherwise,} \end{array}\right) \end{array}$$

where Pr_*i*_ ∈ [0,1] is a probability sampling variable employed to keep the operators of LF and HBA comparable while amending the solutions.

The following step is to assess whether or not the stop conditions have been satisfied, and if so, the best solution will be provided. If this occurs, the upgrading operation is restarted from the start. The pseudocode for the proposed LFHBA is presented in Algorithm 2.

### 4.3. 6G-Enabled IoMT Framework

**Figure 4:**
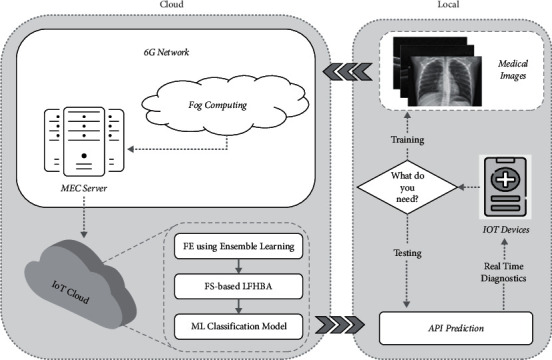
The developed 6G-enabled IoMT framework diagram.

Figure 4 depicts the proposed 6G-enabled IoMT system. The IoT devices capture medical images initially, and if the user's goal is to train our system, the input medical images could well be delivered via a 6G network. Fog computing and multi-access edge computing (MEC) servers are major elements of the 6G network design since they reduce latency and bandwidth usage for commonly used software across a broad scope of various terminals. The gathered information from the MEC could perhaps be sent to a cloud computing provider. In cloud computing, the three core operations remain in place. The characteristics of the DL architecture were recovered in the first step, as explained in Section 4.1. As a second step, as shown in Section 4.2, we utilize the improved HBA depending on LF, named LFHBA, to identify the relevant features. Furthermore, after the classification has been trained, it can be deployed over many API prediction units, reducing transmission costs.

If the specialist's purpose is to evaluate the disease of the captured image, the classification algorithm is used in API prediction tools. Time was reduced because the API allows the platform's approved training to predict without the need for retraining, thus decreasing Internet communications. Ultimately, the sender will be provided with the most recent diagnosis as well as many assessment measures, such as accuracy, to support the software's projections.

## 5. Experimental Results and Discussion

In this section, we will briefly go through the details about the datasets used in this research, followed by evaluation metrics. Next, the effectiveness of optimization feature selection methods is given and studied. Finally, we conclude this section by comparing our proposed method with the state-of-the-art methods.

### 5.1. Dataset

**Figure 5:**
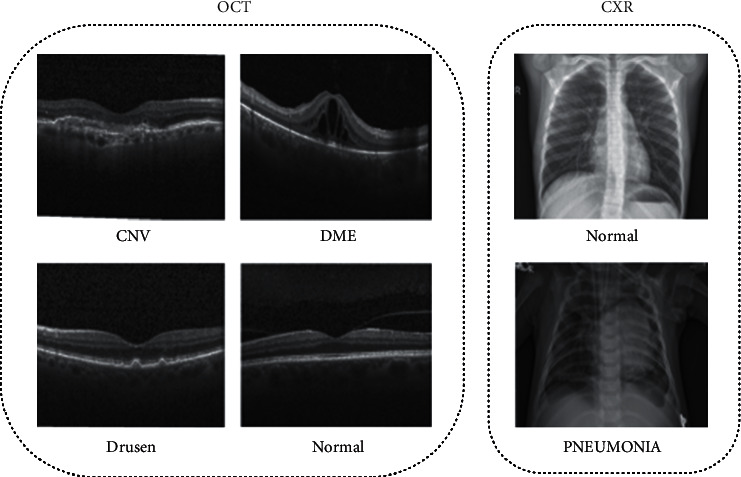
Example medical image samples for classification task from the two selected databases. (a) The optical coherence tomography (OCT) images. (b) The chest X-ray (CXR) images.

Our experiments were evaluated on the chest X-ray (CXR) images and the retinal optical coherence tomography (OCT) images. The two datasets were applied in Guangzhou Women and Children Medical Center [46].  Figure 5, for instance, displays a selection of images from the chosen databases. In the CXR image (pneumonia) dataset, which is publicly available at https://www.kaggle.com/paultimothymooney/chest-xray-pneumonia, there are in total 5856 normal and pneumonia X-ray images. To provide a fair comparison platform for different systems, the training set, validation set, and the test set have been partitioned beforehand. Examples of normal and pneumonia samples can be seen in the right of Figure 5. Details about dataset1 are shown in Table 1.

Other data used in this research consisted of 84,484 OCT B-scans collected from 4,686 patients at the Shiley Eye Institute of the University of California, San Diego (UCSD), https://www.kaggle.com/paultimothymooney/kermany2018. All of the pictures are classified into four types: Drusen, CNV, DME, and Normal, with the corresponding numbers of 8866, 37455, 11598, and 26565. These pictures (collected from Heidelberg Engineering, Spectralis OCT, Germany) were all chosen retrospectively from a sample of older patients with no age, gender, or ethnicity restrictions. According to the company's program and guidelines, the ultimate OCT images are produced using a horizontally foveal slice from the vital picture format. Additionally, this dataset includes 84,484 OCT B-scans, 968 test images, and 83,516 training images. To be more specific, the training set contains 37,213 CNV, 11,356 DME, 8,624 Drusen, and 26,323 Normal images, whereas the testing set has 242 CNV, 242 DME, 242 Drusen, and 242 Normal images. Additional information on the database is available at [46].

### 5.2. Performance Metrics

The precision, recall, F1-score, accuracy, and balanced accuracy measures are utilized to evaluate the developed approach for recognizing medical images. Let true positive (TP) denote the number of adequately recognized images. Also, false positive (FP) denotes the collection of images that have been incorrectly classified. It is the opposite for true negative (TN). Finally, false negative (FN) denotes the number of images erroneously classified.

In equation (15), precision is measured as the ratio of exact data that conforms to specified characteristics. The recall is measured as the ratio of actual statistics to quantities which should have been explicitly anticipated, as introduced in equation (16). The F1-score, in equation (17), is an indication of imbalanced data between recall and precision. The amount produced across all expected amounts is known as accuracy (equation (18)). Balanced accuracy is defined as the average accuracy achieved across all categories, as shown in equation (19).

(15) $$\begin{array}{c} \text{Precision}=\frac{\text{TP}}{\text{TP}+\text{FP}}, \end{array}$$

(16) $$\begin{array}{c} \text{recall}=\frac{\text{TP}}{\text{TP}+\text{FN}}, \end{array}$$

(17) $$\begin{array}{c} \text{F}1-\text{score}=\frac{2\times \text{precision}\times \text{recall}}{\text{precision}+\text{recall}}, \end{array}$$

(18) $$\begin{array}{c} \text{accuracy}=\frac{\text{TP}+\text{TN}}{\text{TP}+\text{TN}+\text{FP}+\text{FN}}, \end{array}$$

(19) $$\begin{array}{c} \text{balanced accuracy}=\frac{1}{2}\times \left[\begin{array}{c} \frac{\text{TP}}{\text{TP}+\text{FN}}+\frac{\text{TN}}{\text{FP}+\text{TN}} \end{array}\right]. \end{array}$$

### 5.3. Results and Analysis

This section summarizes and discusses the findings of experiments conducted to test the proposed FS optimization method. We begin by evaluating our strategy against other meta-heuristic optimization approaches. After that, the support vector machine (SVM) classifier was assessed against each other. Furthermore, this is followed by a comparison with other existing medical imaging categorization systems with different transfer learning models, including DenseNet, MobileNet, and ensemble model. We now have comparisons for recall, precision, F1-score, balanced accuracy, and accuracy. Lastly, it was compared to previously published methods.

Using the proposed FS method, the results are summarized in the next subsection. For the purpose of evaluating our approach's efficacy, we evaluated it against nine other well-known techniques. The meta-heuristic optimizers include grey wolf optimization (GWO) [47], Aquila optimizer (AO) [43], hunger games search (HGS) [48], arithmetic optimization algorithm (AOA) [49], whale optimization algorithm (WOA) [50], firefly algorithm (FFA) [51], and honey badger algorithm (HBA) [22].

These optimization algorithms are assessed using different metrics to solve complex numerical issues of optimization. Due to the unpredictable nature of measure of performance issues, the dimensions of both datasets were decreased to 30 rows and the number of iterations was 1000 in all trials. The greater the number of search agents, the more likely it is to find the global optimum. The sample value is fixed to 50 for all tests. The number of search agents can be lowered to solve the costly problem.

#### 5.3.1. Results of FS Methods

Multiple metrics are utilized to assess the effectiveness of various optimization strategies. F1-score was used to compare the results of each technique. Results from the CXR and OCT datasets can be found in Tables 2 and 3, respectively. Bolded findings are the most accurate in these tables. According to these results, the ensemble model-based LFHBA outperforms GWO, AO, HGS, FFA, WOA, HBA, and AOA.

For the CXR dataset, the results of the proposed method and other optimizers are shown in Table 2. The DenseNet, MobileNet, and ensemble models have been combined on the eight optimization algorithms in the table. According to the table, merging the LFHBA algorithm with ensemble model surpassed other algorithms by 87.10% in terms of the accuracy score. The AO and HGA optimizers came in the second level with 86.22%. HBA is then used to get the same outcome as GWO (i.e., 86.06%). It is 85.10% for the WOA. AOA and FFA had the worst score, with 84.94%, while our proposed algorithm had the best results on the precision metric, with 88.56% of the vote. 88.38% was the second-best result, which belongs to the HGS algorithm. There was one other algorithm AO that performed poorly, with 87.17%. Recall results were better when using the LFHBA algorithm, which had the best outcomes. The AO and HGS both have the same recall (i.e., 86.22%). With 86.06% of the vote, they are followed closely by the GWO and HBA. 85.10% was reached by the WOA. Finally, the FFA and AOA algorithms have a worse outcome of 84.94%. The proposed LFHBA also outperformed other algorithms on F1-score, with 86.19%. The AO optimizer came in the second level with 85.47%. The HGS optimizer came in the third level with 85.41%. Finally, the FFA gets the poorest performance with 83.96% of the population. In the LFHBA algorithm for the balanced accuracy, 82.82% accuracy was attained. In comparison, AO was ranked second (81.97%). With 81.79%, HGS algorithm is next in line. Only FFA achieved a score of 80.17%, which achieved the worst performance.

The proposed LFHBA algorithm outperformed other optimization techniques on the OCT dataset, as seen in Table 3. The accuracy of the LFHBA algorithm for the ensemble model classifier was 94.32%, the best performance. At the same time, the AO and HBA optimizers were at the second level, with 93.80%. With 93.70% of the vote, the AOA algorithm follows the preceding two. Finally, FFA achieved a score of 93.29%, which achieved the worst performance. For the precision measure, our developed LFHBA approach achieved a score of 94.93%. The AO and HBA algorithms follow our algorithm, which has a 94.55% rating. It was 94.48% for the AOA algorithm to keep up with them. 94.26% and 94.19% are the relative percentages for the WOA and HGS algorithms, respectively. As to improve comparison, the recall metric for the ensemble model was 94.32% for LFHBA, the proposed algorithm with the maximum effectiveness. 93.80% was the second-best outcome, which is consistent with the results obtained by the two optimizers (i.e., AO and HBA). Our developed LFHBA algorithm was the best optimizer, with a F1-score of 94.30%. LFHBA is preceded by the AO and HBA methods, which have a combined score of 93.78%. Next, AOA, FFA, GWO, and WOA have 93.68%, 93.57%, 93.45%, and 93.35%, respectively. Finally but not latest, HGS got the poorest performance with 93.26%. There was a 94.32% balanced accuracy of the LFHBA algorithms, which was the best performance. AO and HBA were in the second level with 93.80%. Regarding the AOA's and the FFA's performance in the third and fourth levels, respectively, they scored 93.70% and 93.60%. GWO is behind them with 93.49%. HGS scored 93.29%, the lowest possible score.

**Figure 6:**
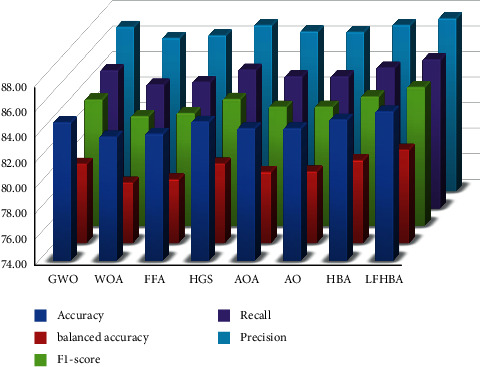
The average results of three models on different FS optimizers in CXR dataset.

**Figure 7:**
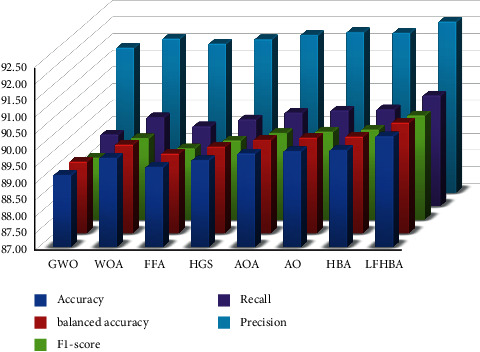
The average results of three models on different FS optimizers in OCT dataset.

From a different viewpoint, the average of the three models (i.e., DenseNet, MobileNet, and ensemble model) using the eight feature selection optimizers investigated the two chosen datasets: CXR and OCT, which are displayed in Figures 6 and 7, respectively. As shown in Figure 6, the overall average accuracy on the CXR dataset is approximately 85.68% for the LFHBA optimizer, whereas the HBA classification model comes in second with 85.04%. A higher level of HGS (with 84.88%) results is preferable to the GWO classification algorithm (i.e., 84.83%). Furthermore, the AOA and AO algorithms outperform the FFA algorithm, with a success rate of 84.35% for AOA and AO and 93.92% for FFA. Besides, the WOA beats other classifiers in terms of accuracy (with 83.71%). From a different point of view, the overall balanced accuracy of the LFHBA algorithm is the best (81.34%). It is preceded by the HBA (80.51%), the GWO (80.23%), and the HGS (80.24%). The AO achieved 79.62%, while the AOA achieved 79.59%. Finally, the FFA and WOA algorithms obtained 79.01% and 78.76%, respectively. Additionally, the average F1-score of the three models beat the LFHBA method by about 84.88%; the HBA algorithm takes second place with 84.14%. Furthermore, the GWO method outperforms the HGS algorithm, with a success rate of 83.91% for XGB and 83.95% for HGS. The HGS algorithm delivers superior results than AO, AOA, FFA, and WOA optimizer, with 83.35%, 83.34%, 82.83%, and 82.59%, respectively. Furthermore, the LFHBA beats other optimizers in terms of recall. To be more specific, the LFHBA achieved 85.87%, while the HBA achieved 85.04%. Finally, the HGS, GWO, AOA, AO, FFA, and WOA algorithms obtained 84.88%, 84.83%, 84.35%, 84.35%, 83.92%, and 83.71%, respectively. In terms of precision measure, the LFHBA algorithm delivers superior results than HBA, HGS, GWO, AOA, AO, FFA, and WOA, with 87.03% 87.02%, 86.88%, 86.52%, 86.48%, 86.21%, and 86.01%, respectively.

On the OCT dataset, as displayed in Figure 7, the overall accuracy of our proposed algorithm is the best (90.33%). It is preceded by the HBA (89.91%), the AO (89.88%), and the AOA (89.81%). Furthermore, the WOA beats the other optimizers. To be more specific, the WOA achieved 89.67%, while the HGS achieved 89.60%. Finally, the FFA and GWO algorithms obtained 89.39% and 89.15%, respectively. In addition, the balanced accuracy of the ten optimization techniques beat the LFHBA method by a total of about 90.33%; the HBA algorithm takes second place with 89.91%. Furthermore, the AO method outperforms the AOA algorithm, with a success rate of 89.88% for AO and 89.81% for AOA. It is preceded by the WOA (89.67%), the HGS (89.60%), the FFA (89.40%), and the GWO (89.15%). The overall average F1-score is approximately 90.13% for the LFHBA classifier, whereas the HBA classification model comes in second with 89.71%. The results of AO results (with 89.65%) are preferable to the AOA algorithm. It is preceded by the WOA (89.45%), the HGS (89.38%), the FFA (89.15%), and the GWO (88.86%). In terms of recall measure, the LFHBA algorithm delivers superior results than HBA, AO, AOA, WOA, HGS, FFA, and GWO algorithm, with 89.91%, 89.88%, 89.81%, 89.67%, 89.60%, 89.39%, and 89.15%, respectively. Additionally, the LFHBA beats other optimizers in terms of precision. To be more specific, the LFHBA achieved 92.14%, while the AO and HBA achieved 91.83% and 91.80%, respectively. Finally, the AOA, WOA, HGS, FFA, and GWO algorithm obtained 91.74%, 91.63%, 91.61%, 91.48%, and 91.36%, respectively.

**Figure 8:**
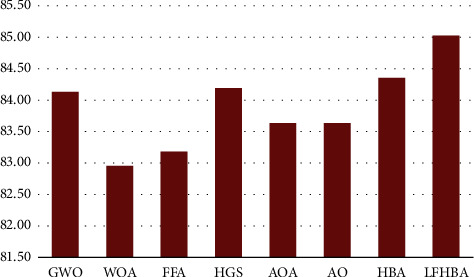
Average results of the five measures on different optimizers in CXR dataset.

**Figure 9:**
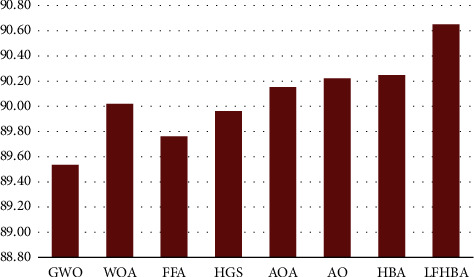
Average results of the five measures on different optimizers in OCT dataset.

The average results of the five measures (i.e., accuracy, F1-score, precision, recall, and balanced accuracy) on the CXR and OCT dataset are presented in Figures 8 and 9, respectively, using different optimization strategies (i.e., the eight optimizers that were introduced before). As seen in Figure 8, the LFHBA played a more critical role than other algorithms. To be more precise, the proposed LFHBA algorithm had an average result of 85.03%, while the HBA had a result of 84.35%. Furthermore, the HGS and GWO algorithms achieved a result of 84.19% and 84.13%, respectively. Then, follows the AOA algorithm, which achieved 83.63%. In all, 83.62% was achieved on the AO optimizer. Other optimizers, FFA and WOA, obtained 83.18% and 82.96%, respectively.

On the OCT dataset, Figure 9 demonstrates that the LFHBA method has a significant influence in selecting features; this is evident across the average of the five metrics. The proposed LFHBA algorithm correctly classifies 90.95% of the testing sample when employing the SVM classification model, more significant than the accuracy of all the other FS optimization methods. Alternatively, the HBA had the second-level outstanding performance at 90.25%. AO was placed in third level. Following these optimizers are the AOA and the WOA, which achieved 90.15% and 90.02%, respectively. Furthermore, the HGS, FFA, and GWO algorithm achieved 89.96%, 89.76%, and 89.54%.

**Figure 10:**
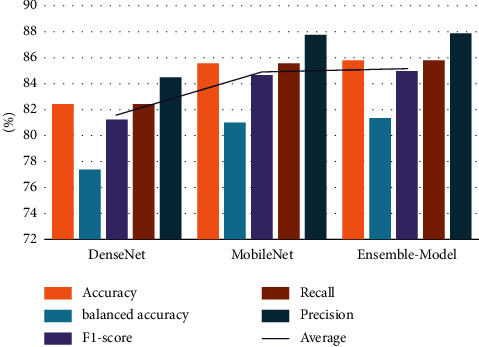
Average results of different optimizers on the three models on the CXR dataset.

**Figure 11:**
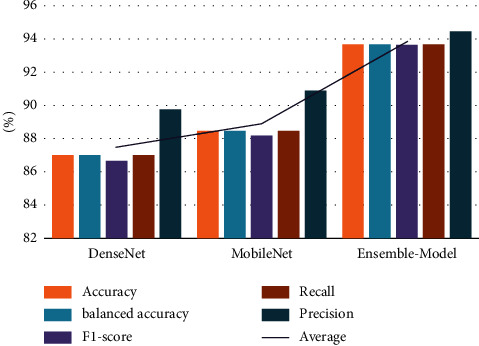
Average results of different optimizers on the three models on the OCT dataset.

To further analysis, the average results of the five measures of different optimizers on the three TL models of the CXR, OCT dataset is introduced in Figures 10 and 11, respectively. In Figure 10, the average results of ensemble model was 85.80%, this model with the maximum effectiveness in term of accuracy score. 85.56% was the second-best outcome, which is consistent with the results obtained by the MobileNet architecture, while DenseNet was the worst in the performance (i.e., 82.43%). Furthermore, the ensemble method achieved the highest results in balanced accuracy, at 81.35%. For all the three networks, MobileNet architecture came in second place with 81.00%. Finally, the DenseNet achieved 77.39%, which was lower than others. The ensemble model was the best optimizer, with a F1-score of 84.96%. The ensemble model is preceded by the MobileNet and DenseNet architectures, which have 84.68% and 81.23%, respectively. In terms of recall measure, the ensemble model delivered superior results than MobileNet and DenseNet, with 85.56% and 82.43%, respectively. Additionally, the ensemble model beats other networks in terms of precision. To be more specific, the ensemble model achieved 87.88%, while the MobileNet achieved 87.77%. Finally, the DenseNet network obtained 84.50%. The overall average of different measures is approximately 85.16% for the ensemble model, whereas the MobileNet model came in second with 84.91%. The DenseNet network had the worst performance across different metrics (at 81.60%).

On the OCT dataset, the average results of different optimizers are displayed in Figure 11. In terms of accuracy measure, the ensemble model had the best performance (93.67%). It is preceded by the MobileNet (88.47%) and the DenseNet (87.01%). In addition, the average balanced accuracy of the eight optimization techniques beat the ensemble method by a total of about 93.67%; the MobileNet model took second place with 88.47%. Furthermore, the DenseNet method achieved 87.01%, which was the worst performance. The overall average F1-score is approximately 93.65% for the ensemble model, whereas the MobileNet model comes in second with 88.18%. Finally, the DenseNet model obtained 86.66%. In terms of recall measure, the ensemble model delivered superior results than the MobileNet and DenseNet models, with 88.47% and 87.01%, respectively. Furthermore, the ensemble model beats other architectures in terms of precision. To be more specific, the ensemble model achieved 94.46%, while the MobileNet and DenseNet achieved 90.88% and 89.76%, respectively. Finally, the average results of the three methods across different measures are obtained. As seen in the figure, the ensemble learning method played a more critical role than other networks. To be more precise, the ensemble model had an average result of 93.82%, while the MobileNet had a result of 88.89%. Finally, the DenseNet achieved 87.49%.

**Figure 12:**
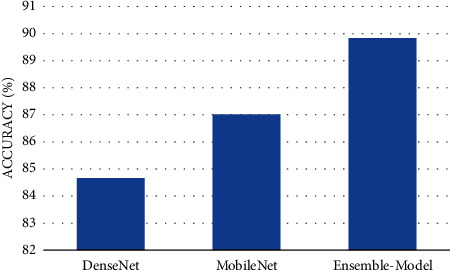
Average accuracy of the three models on the two datasets.

The ensemble model, MobileNet, and DenseNet architectures' average accuracy on the two selected datasets is shown in Figure 12 on various techniques for optimization (i.e., the eight optimizers, introduced before). In the figure, we can see that the ensemble model outperformed other classifiers on the accuracy metric. To be more specific, the ensemble model achieved 89.74% accuracy, whereas the MobileNet achieved 87.02% accuracy. In the end, the DenseNet network achieved 84.72%.

**Figure 13:**
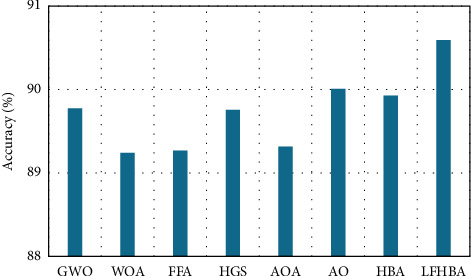
The accuracy of ensemble model on different optimizers on the two selected datasets.

From a clearer point of view on the ensemble model, Figure 13 displays the average accuracy of each feature selection approach on the two datasets, namely: CXR and OCT, from a different perspective. On average, the proposed LFHBA optimizer outperformed the others by about 90.59%; the AO method comes in second with 90.01%. The HBA delivered superior results than GWO and HGS, with 89.78% and 89.67%, respectively. 89.32% of the vote goes to the AOA. After that, the FFA and WOA obtained the lowest results, with average accuracy of 89.27% and 89.25%, respectively.

**Figure 14:**
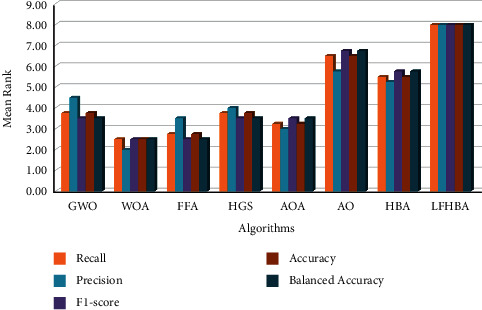
The mean rank of Friedman test.

The statistical value is calculated and ranked by the Friedman (FD) test [52]. The FD test is used to calculate the difference among various approaches.  Figure 14 compares the proposed algorithm to other optimization algorithms. When the proposed method is analyzed in terms of recall, precision, F1-measure, accuracy, and balanced accuracy, it outperforms the others. In terms of recall, the LFHBA has the highest mean ranking of 8, followed by AO which has the mean rank of 6.50. They were followed by HBA, which achieved 5.50. HGS and GWO have nearly identical mean levels, with 3.75. Finally, AOA, FFA, and WOA are lower than the others, with a mean rank of 3.25, 2.75, and 2.50, respectively. According to the FD test results for precision, LFHBA is also better than others, with a mean rank of 8. It was followed by AO and HBA, which achieved 5.75 and 5.25, respectively. On the other hand, GWO and HGS have the same mean level of 4. Lastly, FFA, AOA, and WOA have the lowest mean ranking. Furthermore, we discovered that the LFHBA in terms of the F1-score measure has the best mean rank of 8, and the AO and HBA have the second and third mean ranks of 6.75 and 5.75, respectively. AOA, HGS, and GWO have the same mean levels (i.e., 3.50). Finally, WOA and FFA achieved the lowest mean rank with 2.50. Finally, themean rankaccording to theaccuracy for LFHBA and the AO, HBA, HGS, GWO, AOA, FFA, and WOA optimization algorithms is 6.50, 5.50, 3.75, 3.75, 3.25, 2.75, and 2.50, respectively. According to the FD test results for balanced accuracy, LFHBA is also better than others, with a mean rank of 8. It was followed by AO, which achieved 6.75. HBA has a mean rank level of 5.75, whereas AOA, HGS, and GWO have 3.50. Lastly, WOA and FFA have the lowest mean ranking.

To summarize, for the CXR and OCT datasets, the proposed LFHBA optimization strategy combined with the ensemble model obtained the highest accuracy score.

#### 5.3.2. Comparison with State-of-the-Art Methods

This section compares the proposed method with other state-of-the-art medical image classification techniques.  Table 4 shows the results of a few important methodologies. The development of high-accuracy technology for medical image classification is a major undertaking. It is important to compare our strategy to other models that have been tested on the same datasets. Using CXR and OCT datasets, Table 4 evaluates the performance of several techniques for disease identification.

The CXR dataset was used to compare the various advanced methods for pneumonia detection. In [53], they examined using generative adversarial networks (GANs) to enrich a dataset by producing chest X-ray data samples. For pneumonia diagnosis, Ayan and Ünver [54] employed two well-known convolutional neural network algorithms, Xception and VGG16. In [55], they proposed an automatic transfer learning method based on convolutional neural networks using DenseNet121 pretrained concepts.

To evaluate the developed LFHBA's performance on the OCT dataset, four well-known OCT classification algorithms are tested: the HOG-SVM [56] algorithm efficiently extracts features from the HOG descriptor and subsequently learns a multi-class SVM model for OCT categorization. In [46], transfer learning identifies a conventional CNN by first training it on the ImageNet and then fine-tuning the final convolutional layer on the accessible OCT data. IFCNN [57] utilized a recurrent fusion approach to classify OCT images by using several convolutional features inside CNN. Huang et al. [58] introduced a unique layer guided convolutional neural network (LGCNN) to distinguish between the typical retina and three prevalent macular diseases.

The bottom line is that we can remove superfluous features from high-dimensional medical image representations obtained by convolutional neural network (CNN) using our strategy. However, this framework's fundamental drawback is its complexity, both in terms of time and memory. The following steps include reducing complexity and improving the efficiency of our proposed framework. In the future, other augmentation procedures can be researched to improve our method's efficiency.

The goal of this paper is to present a unique method for improving the performance of the honey badger algorithm (HBA) based on Levy flight (LF). When the HBA is unable to find the optimum solution within a given iteration, it does a more efficient search concentrated on the LF to prevent being trapped in a locally optimal solution. LF search improves the ability to do global and regional searches at the same time.

## 6. Conclusion

This paper details how to classify medical images using 6G technology for IoMT systems. The motivation for this study is that the advantages of 6G over previous generations of wireless connections have recently attracted a lot of interest in the corporate and academic worlds. Moreover, although the medical image classification task has recently grown rapidly, current methods are still not able to achieve good results due to the similarity in the physical features of the image data. Additionally, we have implemented IoMT in our system to assist physicians and patients make fast and advanced diagnosis of diseases worldwide. In order to build our system, it requires training before using cloud center classification techniques. In the training phase, the medical images are collected by IoT devices and communicated to the cloud center. Next, these images are sent to the 6G network to decrease delay and bandwidth consumption. Then, the features of these images were obtained using an ensemble learning model. This model was improved to create more relevant feature vector representations that are beneficial to the medical field. Thus, a unique meta-heuristic technique based on selected features combines the honey badger algorithm (HBA) with Levy flight to pick the important features. The proposed algorithm has a fast convergence velocity, indicating that it avoids capturing in local optimization and effectively balances the exploration and exploitation stages due to the quick classification of thresholds. To evaluate our model, it was tested on the following datasets: CXR and OCT. The findings reveal that the proposed optimization strategy outperforms existing feature selection techniques currently in use. Furthermore, experiments with several cutting-edge methods revealed that the proposed approach is better. However, the proposed model has some crucial problems like time and memory. In the future, we plan to solve these problems by a multi-objective feature selection approach. Also, combining different classification algorithms is also a good research issue since it might help researchers enhance the performance of present methods.

## Figures and Tables

**Algorithm 1:**
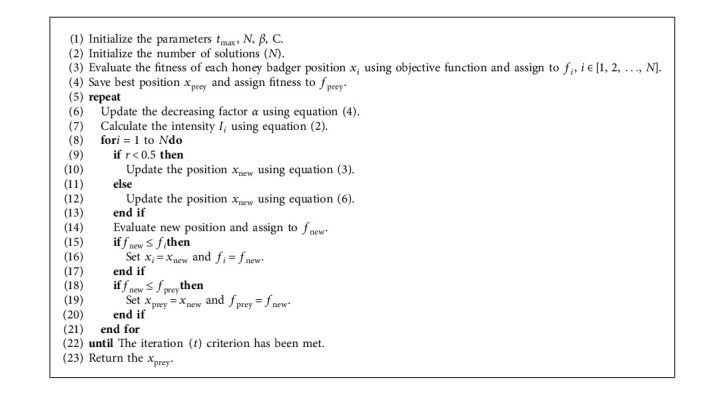
Pseudocode of the proposed HBA.

**Algorithm 2:**
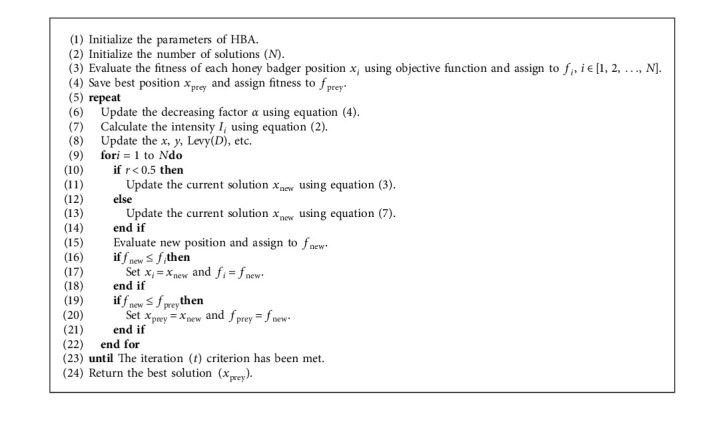
Pseudocode of the proposed LFHBA algorithm.

**Table 1:** Dataset description.

Dataset	Class	Training	Test	Total images
CXR	Normal	1,349	234	1,583
	Pneumonia	3,883	390	4,273
	**Total**	5,232	624	5,856

OCT	CNV	37,213	242	37,455
	DME	11,356	242	11,598
	Drusen	8,624	242	8,866
	Normal	26,323	242	26,565
	**Total**	83,516	968	84,484

**Table 2:** Classification results (%) of each feature selection optimization algorithm on the CXR dataset.

Optimizer	Model	Accuracy	Balanced accuracy	F1-score	Recall	Precision
GWO	DenseNet	82.69	77.86	81.60	82.69	84.48
	MobileNet	85.74	81.24	84.89	85.74	87.89
	Ensemble model	86.06	81.58	85.23	86.06	88.27

WOA	DenseNet	81.25	75.85	79.84	81.25	83.53
	MobileNet	84.78	79.96	83.77	84.78	87.22
	Ensemble model	85.10	80.47	84.17	85.10	87.28

FFA	DenseNet	81.57	76.28	80.22	81.57	83.77
	MobileNet	85.26	80.60	84.33	85.26	87.55
	Ensemble model	84.94	80.17	83.96	84.94	87.33

HGS	DenseNet	82.85	77.99	81.76	82.85	84.74
	MobileNet	85.58	80.94	84.67	85.58	87.94
	Ensemble model	86.22	81.79	85.41	86.22	88.38

AOA	DenseNet	82.21	77.14	80.99	82.21	84.25
	MobileNet	85.90	81.37	85.04	85.90	88.16
	Ensemble model	84.94	80.26	83.99	84.94	87.17

AO	DenseNet	82.21	76.97	80.91	82.21	84.57
	MobileNet	84.62	79.91	83.65	84.62	86.78
	Ensemble model	86.22	81.97	85.47	86.22	88.09

HBA	DenseNet	83.01	78.03	81.87	83.01	85.16
	MobileNet	86.06	81.75	85.28	86.06	87.97
	Ensemble model	86.06	81.75	85.28	86.06	87.97

LFHBA	DenseNet	83.65	78.97	82.66	83.65	85.49
	MobileNet	86.54	82.22	85.78	86.54	**88.61**
	Ensemble model	**87.10**	**82.82**	**86.19**	**87.10**	88.56

**Table 3:** Classification results (%) of each feature selection optimization algorithm on the OCT dataset.

Optimizer	Model	Accuracy	Balanced accuracy	F1-score	Recall	Precision
GWO	DenseNet	85.85	85.85	85.35	85.85	89.05
	MobileNet	88.12	88.12	87.78	88.12	90.74
	Ensemble model	93.49	93.49	93.45	93.49	94.28

MFO	DenseNet	86.67	86.67	86.38	86.67	89.45
	MobileNet	88.53	88.53	88.25	88.53	91.00
	Ensemble model	93.80	93.80	93.78	93.80	94.55

WOA	DenseNet	87.19	87.19	86.86	87.19	89.84
	MobileNet	88.43	88.43	88.15	88.43	90.79
	Ensemble model	93.39	93.39	93.35	93.39	94.26

FFA	DenseNet	86.26	86.26	85.88	86.26	89.46
	MobileNet	88.33	88.33	87.99	88.33	90.57
	Ensemble model	87.19	87.19	86.87	87.19	89.78

HGS	DenseNet	87.19	87.19	86.87	87.19	89.78
	MobileNet	88.33	88.33	88.02	88.33	90.87
	Ensemble model	93.29	93.29	93.26	93.29	94.19

AOA	DenseNet	86.98	86.98	86.65	86.98	89.66
	MobileNet	88.74	88.74	88.49	88.74	91.08
	Ensemble model	93.70	93.70	93.68	93.70	94.48

AO	DenseNet	87.40	87.40	87.03	87.40	89.95
	MobileNet	88.43	88.43	88.13	88.43	90.98
	Ensemble model	93.80	93.80	93.78	93.80	94.55

HBA	DenseNet	87.50	87.50	87.17	87.50	90.01
	MobileNet	88.43	88.43	88.18	88.43	90.85
	Ensemble model	93.80	93.80	93.78	93.80	94.55

LFHBA	DenseNet	87.71	87.71	87.44	87.71	90.30
	MobileNet	88.95	88.95	88.66	88.95	91.19
	Ensemble model	**94.32**	**94.32**	**94.30**	**94.32**	**94.93**

**Table 4:** Accuracy results of the state-of-the-art methods (the best results for each item are labeled in bold).

Dataset	Model	Accuracy (%)	Reference
CXR	DGGAN	84.19	[53]
	VGG16	87.00	[54]
	DenseNet121	86.80	[55]
	**EL** + **LFHBA**	**87.10**	**Ours**

OCT	HOG-SVM	77.80	[56]
	IFCNN	87.30	[46]
	LGCNN	89.90	[58]
	**EL** **+** **LFHBA**	**94.32**	**Ours**

## Data Availability

The data used to support the findings of this study are available from the corresponding author upon request.
